# Genomic Structure Predicts Granulovirus Resistance Without a Detectable Life‐History Trade‐Off in *Plodia interpunctella*

**DOI:** 10.1111/mec.70563

**Published:** 2026-09-28

**Authors:** P. Signe White, Johnathan Lo, Jonathan Barajas, Hailey Burnsed, Noah Parker, Zucel Rodriguez, Natalie Walzer, Peter H. Sudmant, Mike Boots

**Affiliations:** ^1^ Department of Integrative Biology University of California, Berkeley Berkeley California USA

**Keywords:** genome‐wide association, genomic architecture, granulovirus, host‐pathogen interactions, inbred lines, life‐history trade‐offs, pathogen resistance, *Plodia interpunctella*

## Abstract

Understanding the genetic architecture of pathogen resistance and its relationship to host life‐history traits is a fundamental problem in evolutionary biology, yet the conditions under which resistance costs manifest as detectable trade‐offs remain poorly understood. Here, we examine resistance in *Plodia interpunctella* (Indian meal moth) to its granulovirus (PiGV) across 12 inbred lines maintained without deliberate pathogen exposure, alongside an independently assembled outbred stock population as a genomic reference. We quantify resistance using LD50 values from dose–response assays, summarize developmental life‐history variation using a composite principal‐component axis derived from pupal weight, development time and growth rate, and evaluate genomic structure and window‐level associations with resistance. Resistance varied approximately two‐fold across inbred lines. Neither the composite life‐history axis nor any individual life‐history trait was significantly correlated with LD50. In contrast, genomic PC1, the primary axis of CDS‐restricted genomic differentiation, was significantly associated with resistance (*r* = −0.65, *p* = 0.022), whereas overall pairwise distance was not. No individual PCA‐LD50 window survived multiple‐testing correction; instead, resistance‐associated signal was distributed broadly across the genome, and convergent signal spanning five chromosomes showed no enrichment for genes with known immune functions. This pattern is consistent with a diffuse, potentially polygenic basis for resistance, although the modest number of lines limits locus‐level inference. Together with prior work demonstrating resistance‐growth trade‐offs in this system, our results suggest that resistance can be genomically structured without detectable coupling to the developmental life‐history variation measured here, and that expression of resistance costs depends on genetic and environmental context.

## 1 Introduction

Resistance to infection often incurs fitness costs—such as reduced growth rate, extended development time or compromised reproduction—that shape organismal adaptation under pathogen pressure (Adamo 2004; Bartlett et al. 2018; Boots and Begon 1993; Kraaijeveld and Godfray 1997; Lenski and Bouma 1987; Schmid‐Hempel 2005). These trade‐offs have been documented across taxa, from plant systems where resistance and tolerance incur fitness costs that constrain adaptation (Simms and Triplett 1994), to insect host‐pathogen systems where experimental evolution has repeatedly demonstrated that increased resistance comes at the cost of slower development or reduced competitive ability (Boots 2011; Boots and Begon 1993; Kraaijeveld and Godfray 1997). Yet while the existence of resistance costs is well‐established, we understand much less about the genetic architecture underlying these trade‐offs or the conditions under which they manifest. Resource allocation theory predicts that costs of immunity are most apparent when resources are genuinely limiting (Lochmiller and Deerenberg 2000; Sheldon and Verhulst 1996) and empirical work in *Plodia* has confirmed that trade‐offs are context‐dependent. Roberts et al. (2020) showed that resistance‐growth rate correlations depend strongly on nutritional environment. Visher et al. (2022) demonstrated that resource quality, historical contingency and the timescale of selection all shape whether trade‐offs are detectable. Bartlett et al. (2020) further showed that directly selecting on development time did not produce a corresponding change in resistance, suggesting a complex, selection‐direction‐dependent relationship between the traits. Together, these findings raise the question of whether resistance trade‐offs with life‐history traits represent a universal genetic constraint, or whether they depend on genotypic and environmental context.

Understanding the genetic basis of pathogen resistance is a fundamental question in evolutionary biology. *Plodia interpunctella* (Indian meal moth) and its granulovirus (PiGV) provide an ideal system for addressing this question. The tractability of *P. interpunctella* for laboratory work makes it an unusually powerful system for dissecting the genetic basis of host‐pathogen interactions. Its short generation time, tolerance of inbreeding and clear binary infection endpoint make resistance straightforward to quantify across genetically distinct lines. This host‐pathogen pair has been extensively studied, with prior work demonstrating clear fitness costs associated with resistance through experimental evolution (Boots 2011; Boots and Begon 1993). Using inbred lines derived from the same ancestral laboratory stock as those examined here, Bartlett et al. (2018) demonstrated that the resistance‐development time trade‐off in this system is both genetic and constitutive, expressed even in populations with no deliberate prior pathogen exposure, satisfying the strict criteria required by theoretical models of resistance evolution (Boots and Haraguchi 1999; Bowers et al. 1994). The resistance‐growth trade‐off in the *Plodia‐*PiGV system has been demonstrated through both experimental evolution and inbred‐line approaches, providing a well‐evidenced phenotypic backdrop against which genomic analyses can be interpreted.

Genome‐wide approaches have begun to reveal that the genetic architecture of antiviral resistance in insects varies considerably by evolutionary context. In *Drosophila melanogaster* , GWAS and QTL mapping identified a small number of common, major‐effect variants associated with resistance to naturally coevolving viruses, while resistance to non‐coevolved viruses showed lower heritability and no detectable major‐effect loci (Cogni et al. 2016; Duxbury et al. 2019; Magwire et al. 2012). In *Bombyx mori* , a GWAS approach identified *NPC1* as a major locus associated with resistance to baculovirus infection (Kong et al. 2022), one of the few genomic characterizations of viral resistance in Lepidoptera. PiGV is also a baculovirus, but belongs to a different genus: it is a granulovirus (*Betabaculovirus*, family *Baculoviridae*) with a circular double‐stranded DNA genome of 112,536 bp (Harrison et al. 2016), whereas the nucleopolyhedrovirus examined by Kong et al. (2022) is an alphabaculovirus. Resistance in the *Bombyx* system has been associated with major‐effect loci and relatively simple inheritance. Whether resistance to PiGV in *P. interpunctella* is similarly governed by one or a few major loci or instead reflects a more distributed genomic architecture remains unknown. PiGV's host, *P. interpunctella*, is a cosmopolitan pest of stored grains, nuts and dried foodstuffs. The prevalence and geographic distribution of PiGV in field populations of *Plodia interpunctella* have not been systematically surveyed. Similar gaps occur for other granuloviruses: even for the intensively studied codling moth granulovirus, natural field occurrence has remained difficult to establish despite extensive diagnostic surveys, in part because infected larvae decay rapidly and are rarely recovered (Zimmermann et al. 2013).

Despite this extensive phenotypic characterization, no genome‐wide characterization of resistance variation exists for this species. The recent availability of a chromosome‐level reference genome (Childers et al. 2021; NCBI RefSeq GCF_027563975.1) now makes this possible, allowing the genetic architecture of resistance to be examined in a system where the phenotypic and evolutionary context is already well documented.

Here, we examine resistance to PiGV across 12 inbred lines of *P. interpunctella* maintained without deliberate pathogen exposure, alongside an independently assembled outbred stock population as a genomic reference. Because sublethal infection can be transmitted vertically in *P. interpunctella* (Burden et al. 2002), we cannot exclude low‐level covert infection in these laboratory lines. However, the lines were maintained without deliberate pathogen exposure or intentional selection for resistance. We therefore interpret the resistance differences examined here as standing genetic variation rather than as responses to experimental pathogen pressure. We quantify resistance using LD50 values derived from dose–response assays, summarize life‐history variation using a composite axis derived by principal components analysis of pupal weight, development time and growth rate, and use genome‐wide window‐based analyses to characterize genomic regions associated with resistance variation. Our central finding is that genomic PC1 is significantly associated with resistance (*r* = −0.65, *p* = 0.022), while the composite life‐history axis is not. Resistance variation is distributed diffusely across the genome, and convergent signal spanning five chromosomes shows no enrichment for genes with known immune function. We discuss the implications of this architecture for host‐pathogen coevolution and for the decoupling of resistance from life‐history variation under the conditions examined here.

## 2 Materials and Methods

### 2.1 Experimental Methods

#### 2.1.1 Study System and Populations

The 12 inbred lines descended from a common outbred laboratory stock but were established and propagated as separate lineages. Each had undergone five generations of strict full‐sibling mating before being maintained for an undocumented number of generations at approximately 50 individuals per generation without deliberate sibling pairing. After inheriting the lines, we performed three additional generations of strict full‐sibling matings, using five parallel mating replicates per line and propagating the most viable replicate each generation, to reinforce inbreeding prior to phenotypic and genomic sampling. At the fourth generation (G4), each line was scaled up by transferring ~50 offspring into each of two replicate pots. Thereafter, the healthier pot was used each generation to seed two new pots with 50 individuals, maintaining small effective population sizes and within‐line homogeneity while minimizing the risk of line loss. The laboratory stock from which the inbred lines were derived is no longer available. An outbred stock population, sourced from multiple household infestations in the Oakland/Berkeley, CA area, was maintained concurrently and served as a genetic and phenotypic reference. The stock population was assembled from two source collections (one household in Oakland, one in Berkeley) and maintained as a single combined outbred population. All populations were maintained on a standardized diet (recipe in Table S1) in incubators set at 27°C with a 12‐h light:dark cycle. Phenotypic assays were conducted between generation 5 and generation 13 depending on the line (Table S2); genomic sampling was conducted during the same window. Relative homozygosity was characterized using ROH coverage.

#### 2.1.2 Life‐History Assays

For each population, 50 male and 50 female fifth‐instar larvae were randomly selected into four 25‐well assay grids with standard food and monitored daily for pupation. Pupae remained in wells for 2 days to harden before being weighed to the nearest mg on a microbalance. Only individuals that successfully eclosed were included in analyses of pupal weight, development time and growth rate; all pupae were retained for eclosion‐rate calculations. Development time (days) was recorded from egg‐laying to pupation. Growth rate was calculated as pupal mass divided by development time (mg day^−1^). Eclosion outcome was also recorded for each individual that pupated, allowing eclosion rate to be calculated as a population‐level trait.

To summarize multivariate life‐history variation across populations, we performed a principal component analysis (PCA) on the three population‐mean life‐history traits: pupal weight, development time and growth rate. Traits were standardized to unit variance prior to PCA (prcomp, scale = TRUE). Life‐history PC1 was retained as a composite life‐history index in downstream analyses on the basis of variance explained and loading structure. Differences in pupal weight, development time and growth rate among the 12 inbred lines and the stock population were tested using separate one‐way ANOVAs, followed by Tukey HSD post hoc comparisons when the omnibus effect was significant.

#### 2.1.3 Infection Assays

A serial dilution of *P. interpunctella* granulovirus (PiGV) was prepared in glucose with Coomassie brilliant blue dye as a feeding marker. PiGV dose was expressed relative to a common working virus stock, with the highest concentration designated as 1× (10^0^), followed by three successive 10‐fold dilutions (10^−1^, 10^−2^, 10^−3^). Each population was tested at all four viral concentrations (75 larvae per concentration) and with a no‐virus control (25 larvae), for a total of *n* = 325 larvae per population. Virus droplets were arrayed on petri dishes; larvae foraged for approximately 2 h, with successful ingestion confirmed by blue coloration of the gut. Larvae were then transferred to 25‐well grids (three grids per dose, one grid for control) and incubated at 27°C for 20 days. Individuals were scored as infected based on their characteristic chalky‐white appearance, caused by the accumulation of viral occlusion bodies within infected cells, which is a standard and unambiguous endpoint for granulovirus infection in lepidopteran larvae (Cory and Myers 2003).

Due to staggered emergence times across populations, each population was assayed on a different day; consequently, day and population identity are confounded and day effects cannot be formally partitioned from population effects. To minimize systematic day‐to‐day variation, virus stock and serial dilutions were prepared from the same frozen aliquots using identical protocols across all assay days. For logistical reasons, some populations had doses split across 2 days; in these cases all handling and incubation protocols were identical.

### 2.2 Statistical Modelling

#### 2.2.1 Infection Modelling

We modelled infection probability as a function of viral dose using a binomial logistic generalized linear model (GLM). The model assumes a logistic distribution for the binary response variable (infected = 1, uninfected = 0), and includes dose, population identity and their interaction as predictors. Statistical significance of each term was assessed using likelihood ratio tests (Analysis of Deviance, Chi‐square). All dose–response and population‐level predictions are derived from this model and are presented in the Section 3.

#### 2.2.2 LD50 Estimation

We estimated population‐specific LD50 values—the viral dose at which 50% of individuals are predicted to become infected—by fitting separate logistic regression models to binary infection data (infected = 1, uninfected = 0) for each population, using the dose.p function in the MASS package in R (v7.3.65). LD50 is our primary measure of resistance throughout, as it integrates both the baseline susceptibility and dose sensitivity of a population into a single, biologically interpretable value and is a standard metric in host‐pathogen studies (Boots and Begon 1993).

To visualize infection dynamics across populations, we additionally applied a log‐logit transformation, plotting log‐odds of infection against log‐transformed dose. This approach enabled estimation and visual comparison of baseline susceptibility (intercept) and dose sensitivity (slope) across populations.

#### 2.2.3 Life‐History and Resistance Correlations

To test whether resistance was associated with life‐history traits across the 12 inbred lines, we calculated Pearson correlations between population‐mean LD50 and each life‐history trait (pupal weight, development time and growth rate), as well as with life‐history PC1. Analyses were conducted across all 12 inbred lines, excluding the stock population, treating each line as an independent observation. To assess whether genomic structure predicted resistance, we correlated LD50 values with scores along the first principal component derived from the genomic PCA (see Section 2.3). To test whether life‐history variation was itself genomically structured, we additionally calculated Pearson correlations between life‐history PC1 and genomic PC1 and PC2. To test whether eclosion rate varied among lines, we compared counts of eclosed versus non‐eclosed pupae across 12 inbred lines using a chi‐square test of independence and tested its relationship with resistance using a Pearson correlation between per‐line eclosion rate and population‐mean LD50. Pearson correlations and associated *p*‐values were calculated in R. We considered associations significant at *p* < 0.05.

#### 2.2.4 Expected Inbreeding From Documented Breeding History

To provide a pedigree‐based benchmark for the documented breeding protocol, we calculated the expected inbreeding coefficient under three consecutive generations of full‐sibling mating completed before subsequent line propagation and sampling. Assuming non‐inbred, unrelated ancestors at the beginning of this pedigree, expected *F* increases to 0.25 after one generation of full‐sibling mating, 0.375 after two generations and 0.50 after three generations (Falconer and Mackay 1996). Because the breeding history preceding these three generations is incompletely documented, we do not attempt to estimate the actual pedigree inbreeding coefficient of each line at sampling; *F* = 0.50 is used only as a conditional benchmark for the documented breeding phase.

#### 2.2.5 Exploratory Clustering of Resistance Phenotypes

As an exploratory complement to the overall correlation analyses, we performed *k*‐means clustering on population‐mean LD50 values to examine whether distinct resistance classes showed different life‐history relationships. Model selection was based on within‐cluster sum of squares and percentage improvement in WSS, both of which supported *k* = 3 as the optimal number of clusters. Full model selection criteria, cluster assignments and within‐cluster correlations are presented in Section 3.

### 2.3 Genomic Methods

#### 2.3.1 Genome Assembly and Variant Calling

Fifth‐instar larvae from each inbred line were sequenced on a NovaSeq X Plus platform (350 bp inserts; ~40×–45× coverage). For each line and for the stock population, DNA was extracted from pools of 2–3 individuals per extraction. Because each inbred line was represented by a single pool of 2–3 diploid individuals, these data sample only 4–6 chromosome copies per locus and therefore do not provide precise estimates of allele frequencies for variants that remain segregating within lines. Genomic analyses should consequently be interpreted as characterizing the sampled pooled genotype of each line rather than exhaustively capturing within‐line genetic variation. In addition, genotype calls from pooled DNA extractions cannot distinguish true within‐individual heterozygosity from allelic differences among the 2–3 pooled individuals; a heterozygous call at any given site may reflect either source. This ambiguity is most consequential at sites outside long ROH tracts, where within‐line variation is more likely to persist. Raw reads were assembled into contigs using MaSuRCA v4.1.2 (Zimin et al. 2013) and scaffolded to the chromosome‐level reference genome for *P. interpunctella* (Childers et al. 2021; NCBI RefSeq accession: GCF_027563975.1; BioProject PRJNA782555) using RagTag v2.1.0 (Alonge et al. 2022). BAM files were produced using BWA‐MEM v0.7.17 (Li and Durbin 2009). Variants were called with bcftools v1.6 using mpileup followed by call ‐mv (Danecek et al. 2021). No additional post‐calling site‐level depth, mapping‐quality or private‐singleton filters were applied; private variants were retained.

#### 2.3.2 Population Genomic Structure

To assess genomic structure among inbred lines, per‐sample variant call format (VCF) files were restricted to coding sequence (CDS) variants by intersecting variant coordinates with CDS intervals, using bedtools intersect v2.31.0 (Quinlan and Hall 2010). Variants were imported into R using vcfR v1.16.0 (Knaus and Grünwald 2017) and analysed with adegenet v2.1.11 (Jombart 2008). Principal components analysis (PCA) was performed on the matrix of genotype calls across all retained CDS variants, and populations were visualized along the first two principal components. Throughout the manuscript, ‘genomic PC1’ and ‘genomic PC2’ refer specifically to these CDS‐restricted, inbred‐only components, distinct from the life‐history PC1 described above, the genome‐wide PCA described in the following paragraph (used only as a robustness check), and the separate PCA incorporating the outbred stock population described below. Pairwise genetic distances (Euclidean distance on genotype calls, coded as 0, 1 or 2 copies of the non‐reference allele, across all CDS variant sites) were computed and visualized as a midpoint‐rooted neighbour‐joining tree, constructed using the nj function in the ape package v5.8 (Paradis and Schliep 2019).

As a robustness check on the restriction to CDS variants, we repeated the pairwise distance calculation, neighbour‐joining tree construction, Mantel test and genomic PCA using genome‐wide variants extracted directly from the same multi‐sample VCF (bcftools v1.16 query, no CDS restriction), retaining only sites with a complete genotype call across all 12 lines (1,717,334 of 4,380,423 genome‐wide variants; approximately 6‐fold more variants than the CDS‐restricted analysis). We additionally confirmed that a standard variant‐quality filter (QUAL ≥ 30) had negligible effect on this result (correlation between filtered and unfiltered genome‐wide distance matrices = 1.00).

To incorporate the outbred stock population, VCF files from all 84 pooled stock samples and the 12 inbred‐line samples were merged using bcftools v1.16 and filtered to CDS variants as above. A combined PCA was performed on 194,030 CDS variants across all samples. Each stock sample comprised pooled DNA from 2 to 3 individuals per extraction; the inbred‐line samples are likewise generated from pools of 2 to 3 individuals. Pooling is expected to be more consequential for the genetically heterogeneous outbred stock, but it also introduces uncertainty into the genomic representation of inbred lines where residual within‐line variation remains. The stock pools, by contrast, combine individuals from a genetically heterogeneous outbred population, so pooled genotype calls conflate within‐individual heterozygosity with the allelic differences among individuals. Results from stock genomic analysis should therefore be interpreted as pooled population‐level observations rather than individual genotypes. The combined PCA serves to place genomic differentiation among inbred lines in the context of an independently maintained outbred reference population and to assess whether the stock samples recover the colony's known dual‐source structure, rather than to test genotype–phenotype associations directly; such associations are therefore restricted to the 12 inbred lines. To test whether genomic structure predicted resistance phenotypes, we calculated Pearson correlations between genomic PC1 scores and population‐mean LD50 across all 12 inbred lines. To test whether overall genomic relatedness, rather than a specific axis of genomic structure, predicted resistance similarity, we additionally performed a Mantel test comparing the pairwise genomic distance matrix (the same matrix used to construct the neighbour‐joining tree) against a pairwise matrix of absolute LD50 differences among the 12 inbred lines. A one‐sided alternative hypothesis (positive association between genomic and resistance distance) was tested, with significance assessed using 9999 permutations (mantel function, vegan package v2.7.5).

#### 2.3.3 Window‐Based Genomic Association Analyses

To characterize genomic regions associated with viral resistance, we applied two complementary window‐based approaches across the genome. Chromosome sizes were extracted from the merged VCF header and used to generate sliding windows of 250 kb with 125 kb steps using bedtools makewindows, excluding unplaced scaffolds (NW_contigs). Per‐window VCF files were extracted from the merged inbred VCF using bcftools view, yielding 2232 windows across 31 chromosomes. This window size was selected following empirical estimation of linkage disequilibrium (LD) decay across the genome (pairwise *r* ^2^ between SNPs up to 2 Mb apart), which remained substantial even at long distances (*r* ^2^ = 0.63 at < 50 kb, still 0.26 at 2 Mb). Windows were restricted to complete 250 kb intervals containing at least 100 variants, retaining 2164 of 2232 windows (97%); truncated chromosome‐end windows and windows with fewer than 100 variants (20–50‐fold below the genome‐wide median of 2077 SNPs per window) were excluded, as the association statistics described below are unstable when computed from few markers or non‐comparable window widths.

First, local PCA was performed in each retained window using SNPRelate v1.38 (Zheng et al. 2012). For each window, the first principal component (PC1) score was extracted for each inbred line and correlated with population‐mean LD50 using Pearson correlation, yielding a window‐level association statistic (PCA‐LD50 approach). The proportion of variance in LD50 explained by each window (PVE) was calculated as the squared correlation coefficient (*r* ^2^); the cumulative distribution of window‐level PVE across all retained windows was used to characterize how diffusely resistance‐associated signal was distributed across the genome. *p*‐values were transformed as −log_10_(*p*) and plotted as a Manhattan plot; multiple testing was corrected using a Bonferroni threshold (*α* = 0.05/2164 retained windows). To test whether the diffuse PVE distribution could reflect sampling noise alone given the small number of lines, we permuted LD50 across the 12 lines 1000 times, recomputing the window‐level PVE distribution under each permutation to generate a null envelope.

Second, Weighted *Z*‐score Analysis (WZA; Booker et al. 2024) was applied across the same retained windows to aggregate SNP‐level association signals into window‐level scores as a distinct, complementary approach to the window‐level PCA analysis. For each SNP passing a minor allele frequency threshold of 0.05, we computed a per‐SNP association statistic via Pearson correlation between genotype (coded as 0, 1 or 2 copies of the non‐reference allele) and population‐mean LD50 across the 12 inbred lines. The resulting raw *p*‐values were converted to empirical *p*‐values by ranking them across all SNPs, and these were in turn converted to *Z*‐scores. Window‐level WZA scores (Z_W) were computed as the heterozygosity‐weighted sum of SNP‐level *Z* scores, then normalized by SNP count using a Gamma generalized linear model with a log link, which constrains the predicted dispersion to remain strictly positive; the resulting mean absolute deviation was converted to a standard deviation (*σ* = (mean absolute deviation) × √(*π*/2)) for normalization. Because SNPs within windows remain linked, WZA was treated as a ranking statistic rather than a formal window‐level significance test; windows were therefore ranked directly by the absolute value of the normalized score |Z_Wnorm_|. Allele frequencies for WZA were calculated across all inbred lines. QQ plots were examined as diagnostics of the distribution of test statistics for both approaches (Figure S7).

We defined convergent regions as windows ranking in the top 1% or top 2% by PVE, with supporting evidence from the WZA ranking (also top 1% or top 2%, respectively). This joint threshold was pre‐specified prior to inspection of which genes fell within these windows and is reported as a ranking criterion rather than a formal significance test; results of genome‐wide multiple‐testing correction are reported separately. Because linkage disequilibrium in this dataset remains substantial at distances of at least 2 Mb, convergent windows within this distance of one another on the same chromosome were not treated as independent signals.

To test whether convergent regions were enriched for genes with known immune function, we intersected the peak window within each of the 10 convergent regions with the *P. interpunctella* genome annotation (GCF_027563975.1) using bedtools intersect, retaining gene‐level features and searched gene descriptions against two keyword sets: a strict set restricted to canonical immune pathway genes, and a broader set additionally including proteostasis, autophagy, oxidative stress, stress‐response, apoptosis and protease/detoxification‐related terms (Table S6). Keyword sets were finalized after the peak‐window gene list had already been compiled; both were deliberately drawn broadly from canonical insect immunity pathways, including many terms absent from the observed gene list, so that any unconscious bias in keyword selection would work against detecting enrichment rather than toward it. Observed keyword‐match counts were compared against a null distribution generated by drawing the same number of windows at random from the full set of retained windows, 10,000 times.

#### 2.3.4 ROH Quantification

To quantify long homozygous sequence in the inbred lines and provide a descriptive comparison with the outbred reference stock, runs of homozygosity (ROH) were identified using bcftools roh v1.16. As population‐specific allele frequencies were unavailable, the software default allele frequency of 0.4 was applied. Both inbred‐line and stock sequencing libraries were generated from pools of 2 to 3 individuals. In pooled samples, differences among individuals can appear as heterozygous genotype calls and alter inferred ROH tracts. This issue is expected to be especially consequential in the genetically heterogeneous stock pools but may also affect inbred‐line estimates where residual within‐line variation remains. ROH were identified separately from the merged inbred VCF and the merged stock VCF. For each pooled sample, the total length of all ROH segments was summed and expressed as a percentage of the total assembled genome size (291 Mb). We therefore interpret stock ROH coverage as a descriptive pooled‐sample comparison rather than an individual‐level genomic inbreeding coefficient. For the inbred lines, ROH coverage similarly provides a pooled‐sample measure of long homozygous sequence that can be used to compare relative homozygosity among lines, but it should not be interpreted as a precise individual‐level inbreeding coefficient. Although ROH coverage is calculated as total ROH length divided by genome length—the same ratio conventionally denoted *F*_ROH_ for individually sequenced diploid genomes—we refer to this quantity as ROH coverage throughout because our genomic samples comprise pools of 2–3 individuals.

## 3 Results

### 3.1 Genomic Structure Among Inbred Lines

The neighbour‐joining tree of genomic relatedness revealed substantial differentiation among inbred lines (Figure S2). Overall genomic distance was not significantly correlated with pairwise differences in LD50 across all lines (Mantel test, *r* = 0.16, *p* = 0.14, 9999 permutations), indicating that resistance variance is not simply a function of general genomic relatedness. Instead, resistance is specifically associated with genomic PC1, the primary axis of genomic differentiation (*r* = −0.65, *p* = 0.022, Figure 1), suggesting that a particular dimension of genomic structure, rather than overall divergence, underlies resistance variation among these lines. This result was robust to the choice of variant set: repeating the analysis on genome‐wide, complete‐call‐filtered variants yielded a distance matrix significantly concordant with the CDS‐restricted matrix (Mantel *r* = 0.57, *p* < 0.001, 9999 permutations), and preserved both the identification of IL‐17 and IL‐36 as the most closely related pair and IL‐4 as the most genomically divergent line (Figure S2). The relationship between overall genomic distance and LD50 was also consistent under genome‐wide analysis (*r* = 0.10, *p* = 0.27). The association between genomic PC1 and LD50 remained consistent in direction and magnitude under genome‐wide PCA (*r* = −0.57, 95% CI: −0.86, 0.003; Figure S13), though this narrowly missed conventional statistical significance (*p* = 0.052, compared to *p* = 0.022 for the CDS‐restricted analysis), potentially reflecting dilution of the resistance‐associated axis by a much larger proportion of neutral background variation genome‐wide. Principal components analysis of the full dataset including the stock population confirmed that the stock is genomically distinct from the inbred lines, separating clearly along PC1, which explained 12.8% of variance, with PC2 explaining a further 4.8% (Figure 2). Within the stock, two clusters were apparent, consistent with the two source populations used to establish the colony (Oakland and Berkeley). The inbred lines formed a separate, more dispersed cluster on the opposite end of PC1, indicating substantial genomic differentiation among lines.

**FIGURE 1:**
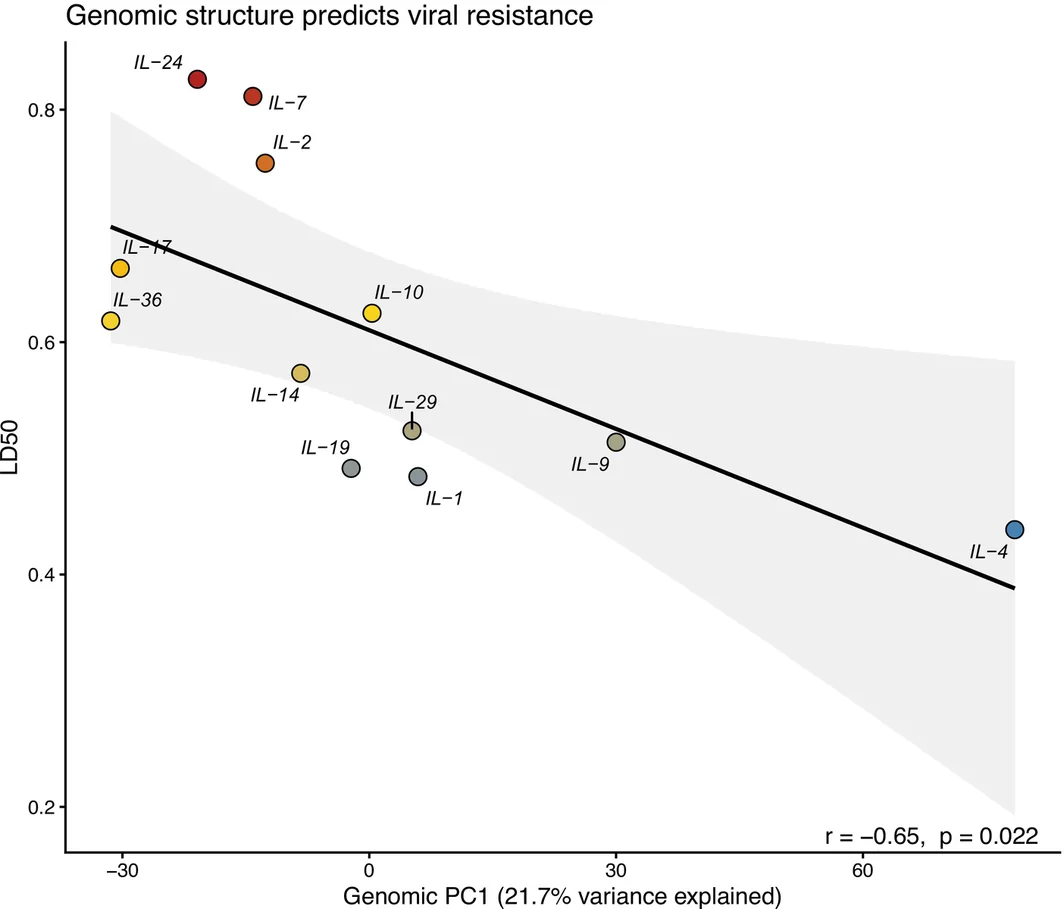
Genomic structure predicts granulovirus resistance across inbred lines of *Plodia interpunctella*. Each point represents one of the 12 inbred lines, plotted by its score along the first principal component of the CDS‐restricted PCA (genomic PC1, explaining 21.7% of total variance) against its population‐mean LD50. Points are coloured by LD50 value (blue = susceptible, gold = intermediate, red = resistant). The solid line shows the linear regression fit and the shaded ribbon indicates the 95% confidence interval. Lines at the negative end of genomic PC1 tended to have higher LD50 values, whereas lines at the positive end tended to be more susceptible. Because the sign of a principal component is arbitrary, this direction should be interpreted only as position along the axis. The negative correlation is statistically significant (*r* = −0.65, *p* = 0.022), indicating that resistance covaries with the primary axis of genomic differentiation among the inbred lines. The independently assembled stock population is excluded because the genomic PC1‐resistance association is defined across the 12 matched inbred‐line genomic samples and line‐level resistance phenotypes.

**FIGURE 2:**
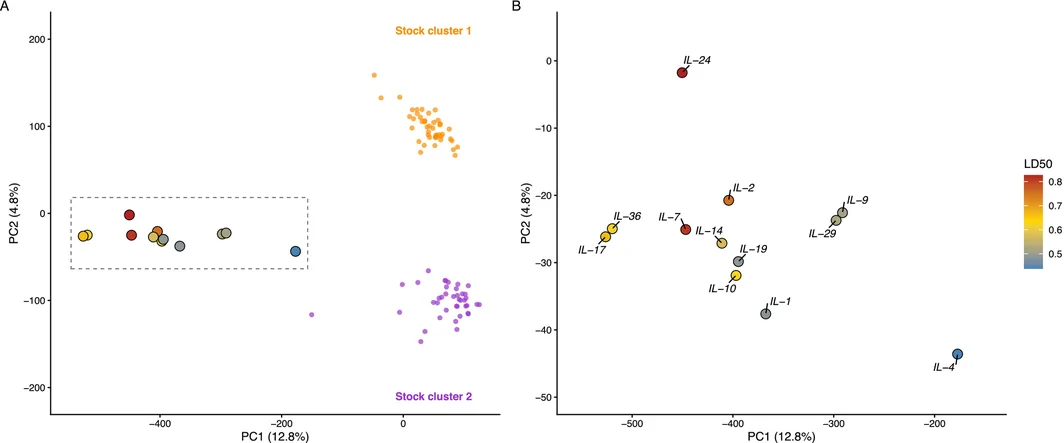
Genomic principal components analysis of inbred lines and stock population of *Plodia interpunctella*. (A) Full PCA plot showing all samples along the first two principal components of the combined PCA performed on 194,030 coding sequence variants. Inbred line points are coloured by LD50 (blue = susceptible, gold = intermediate, red = resistant). Stock population samples form two discrete clusters (cluster 1 = orange; cluster 2 = purple), consistent with their dual‐source origin from two independent household collections. The dashed rectangle indicates the region expanded in panel B. (B) Zoomed view of the inbred line cluster, with lines labelled in italics and coloured by LD50. PC1 explains 12.8% and PC2 explains 4.8% of total variance. Inbred lines cluster tightly together relative to the stock population, reflecting their reduced genomic diversity following inbreeding, and separate clearly from both stock clusters along PC1.

Runs of homozygosity analysis showed substantially greater ROH coverage in the inbred lines than in the outbred stock overall (Figure S3A). Total ROH length ranged from 42.0 Mb (IL‐4) to 93.5 Mb (IL‐17) across inbred lines, corresponding to 14.4%–32.1% of the 291 Mb genome (mean 26.1%). By contrast, stock samples carried a mean of 7.3% of the genome in ROH (range 1.3%–26.6%, *n* = 84), approximately four‐fold less than the inbred line mean (Figure S3B). Only 3 of 84 stock pools (3.6%) reached or exceeded ROH coverage of the lowest inbred line, and none reached the coverage of the highest inbred line, indicating that the distinction between inbred lines and outbred samples remains robust despite this minor overlap. A Wilcoxon rank‐sum test confirmed that the two distributions differ significantly (*W* = 1001, *p* < 0.001; rank‐biserial effect size = 0.99). ROH coverage was positively correlated with LD50 across inbred lines (*r* = 0.68, *p* = 0.016). Because genomic PC1 was also associated with LD50, the independent contributions of genomic structure and differences in homozygosity cannot be resolved in this dataset. We therefore do not interpret the ROH‐LD50 association as evidence that greater homozygosity itself increases resistance.

### 3.2 Resistance Variation and Its Genetic Basis

Infection probability increased with viral dose across all populations, as confirmed by binomial logistic GLM analyses (Figures S4–S6). Dose was the dominant predictor of infection outcome (*χ* ^2^ = 1267.24, df = 1, *p* < 0.001; Table S3). Population identity (*χ* ^2^ = 40.28, df = 12, *p* < 0.001) and the dose × population interaction (*χ* ^2^ = 23.12, df = 12, *p* = 0.027) also contributed significantly, suggesting heterogeneity in dose–response relationships among populations.

Population‐specific LD50 values estimated from separate logistic regression models revealed substantial variation in resistance across inbred lines, ranging from 0.439 (IL‐4, most susceptible; 95% CI [0.311, 0.566]) to 0.826 (IL‐24, most resistant; 95% CI [0.618, 1.034]; Table S4), an approximately two‐fold range. Genomic PC1 was significantly negatively correlated with LD50 (*r* = −0.65, *p* = 0.022; Figure 1), indicating that lines separated along the primary axis of genomic differentiation also differ systematically in their resistance to infection. Lines with higher genomic PC1 scores tended to be more susceptible, while lines at the lower end of PC1 tended to be more resistant. IL‐4, an extreme line on genomic PC1 and the most susceptible line, has notable leverage on this result; excluding it attenuates but does not eliminate the association (*r* = −0.60, *p* = 0.052). As a further robustness check, repeating this analysis using allele‐frequency‐standardized PCA (the convention used by, e.g., EIGENSTRAT) yielded a consistent direction and similar magnitude (*r* = −0.58, *p* = 0.049) to our primary, unstandardized analysis. Together, these checks are consistent with an association between genomic structure and resistance that is nonetheless sensitive to both individual lines and analytic choices, an expected consequence of the sample size available for genomic analysis (*n* = 12).

Genome‐wide window‐based analyses were performed across the 2164 retained windows using both a window‐based PCA approach and the WZA (Figure 3). No individual PCA‐LD50 window survived Bonferroni correction (minimum *p* = 0.000458 against a threshold of 2.31 × 10^−5^), indicating that this analysis did not identify a formally significant individual window. QQ plots confirmed appropriate control of test statistic inflation across the remainder of the genome for both approaches (Figure S7).

**FIGURE 3:**
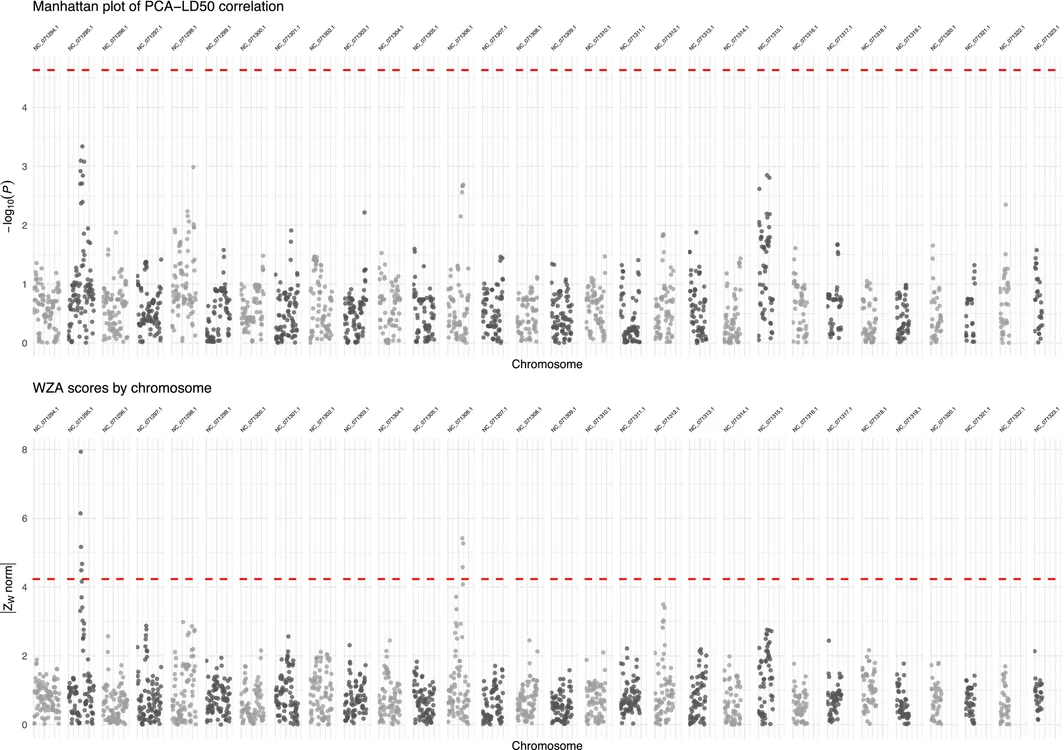
Genome‐wide window‐based analyses of granulovirus resistance in *Plodia interpunctella*. Each point represents one of 2164 retained 250 kb windows (125 kb steps), spanning 30 of the 31 chromosomal scaffolds of the *P. interpunctella* reference genome (GCF_027563975.1); the smallest scaffold, NC_071324.1 (180,911 bp), is shorter than one window and contributes no full‐size windows to the analysed set. Alternating light and dark grey shading distinguishes adjacent chromosomes. (Top) Manhattan plot of the PCA‐LD50 correlation approach: For each window, the first principal component of a local genomic PCA was correlated with population‐mean LD50 across the 12 inbred lines, and the resulting −log_10_(*p*) values are plotted by chromosomal position. The red dashed line indicates the Bonferroni‐corrected significance threshold; no window survives this correction (minimum *p* = 0.000458 against a threshold of 2.31 × 10^−5^). (Bottom) Manhattan plot of Weighted *Z*‐score Analysis (WZA) scores: WZA aggregates SNP‐level genotype‐LD50 associations with LD50 into window‐level scores, accounting for variation in SNP density and allele frequency. The *y*‐axis shows |Z_W,norm|, the normalized WZA score, WZA is used as a ranking statistic rather than a formal window‐level significance test. The red dashed line marks a nominal Bonferroni‐equivalent threshold on the |Z_W,norm| scale, shown for visual reference only and not as a validated significance test. Windows on scaffolds NC_071295.1, NC_071298.1, NC_071306.1, NC_071312.1 and NC_071315.1 meet the pre‐specified joint top 1%/2% ranking criterion by both PVE and WZA, forming 10 contiguous regions (Table S5).

Despite the absence of any significant window, the distribution of window‐level PVE values was not dominated by a small set of genomic regions. The top 1% of windows by PVE contributed 4.7% of summed window‐level PVE across the genome, rising to 29.3% for the top 10% of windows and 55.2% for the top 25%. We treat these percentages as descriptive summaries of the distribution of window‐level *r* ^2^ values rather than as an additive decomposition of phenotypic variance. The observed cumulative PVE curve never exceeded the 95% null envelope at any rank and fell below its lower bound beyond approximately 24% of windows (Figure S12), indicating that window‐level association strength was less concentrated among the highest‐ranked windows and more broadly distributed across the genome than in the permuted datasets. This pattern is consistent with a diffuse, potentially polygenic architecture, but the small number of phenotyped lines limits the ability to distinguish a genuinely polygenic architecture from limited power to resolve stronger loci.

Twenty‐one windows met the pre‐specified joint criterion of ranking in the top 1% or top 2% by both PVE and WZA, forming 10 contiguous regions across five chromosomes: NC_071295.1, NC_071298.1, NC_071306.1, NC_071312.1 and NC_071315.1 (Table S5). Because several of these regions comprise multiple adjacent or overlapping windows within 1.5–2.1 Mb of one another on the same chromosome, approximately the scale over which linkage disequilibrium remains substantial in the dataset (*r* ^2^ = 0.26 at 2 Mb), we report this convergence as signal localized to five chromosomes rather than as 21 or 10 independent hits.

To test whether these convergent regions were enriched for genes with known immune function, we performed a permutation test (10,000 draws) comparing observed keyword matches among the 172 genes within the 10 peak convergent windows against a null distribution of randomly drawn windows from the retained set. Neither the strict keyword set (canonical immune pathway genes; observed = 1, null mean = 1.08, *p* = 0.61) nor the broad set (additionally including proteostasis, autophagy, oxidative stress and protease‐related terms; observed = 8, null mean = 5.96, *p* = 0.27) showed enrichment (Table S6); the strict‐list observed count was in fact below the null expectation. We therefore do not identify specific candidate genes within these regions and interpret the convergent signal as evidence that resistance‐associated signal is distributed across multiple chromosomes rather than concentrated in a small number of mechanistically tractable loci.

### 3.3 Life‐History Variation Among Populations

Populations differed significantly in all three life‐history traits measured: pupal weight (*F*_12,770_ = 23.89, *p* < 0.001), development time (*F*_12,770_ = 26.03, *p* < 0.001) and growth rate (*F*_12,770_ = 9.63, *p* < 0.001; Figure 4). Principal components analysis of these traits yielded a first axis (PC1) explaining 67.8% of total variance, with all three traits loading negatively on PC1 (pupal weight: −0.701, development time: −0.499, growth rate: −0.510), defining a composite axis of developmental and growth variation. Populations with high PC1 scores had low pupal weight, short development time and slow growth rate, while populations with low PC1 scores exhibited heavier pupae, longer development and faster growth. Pupal weight and development time were positively correlated across populations (*r* = 0.74, *p* = 0.004; Figure S1), indicating that these traits covary along a shared axis rather than varying independently. Among individuals that pupated, eclosion rate averaged 0.77 across the 12 inbred lines (range 0.61–0.85) and was 0.87 in the outbred stock (Table S4). Eclosion rate did not differ significantly among inbred lines (*χ* ^2^ = 11.32, df = 11, *p* = 0.42) and was not significantly correlated with LD50 (*r* = −0.49, *p* = 0.11), indicating that pupal‐to‐adult viability was not detectably associated with resistance in this panel.

**FIGURE 4:**
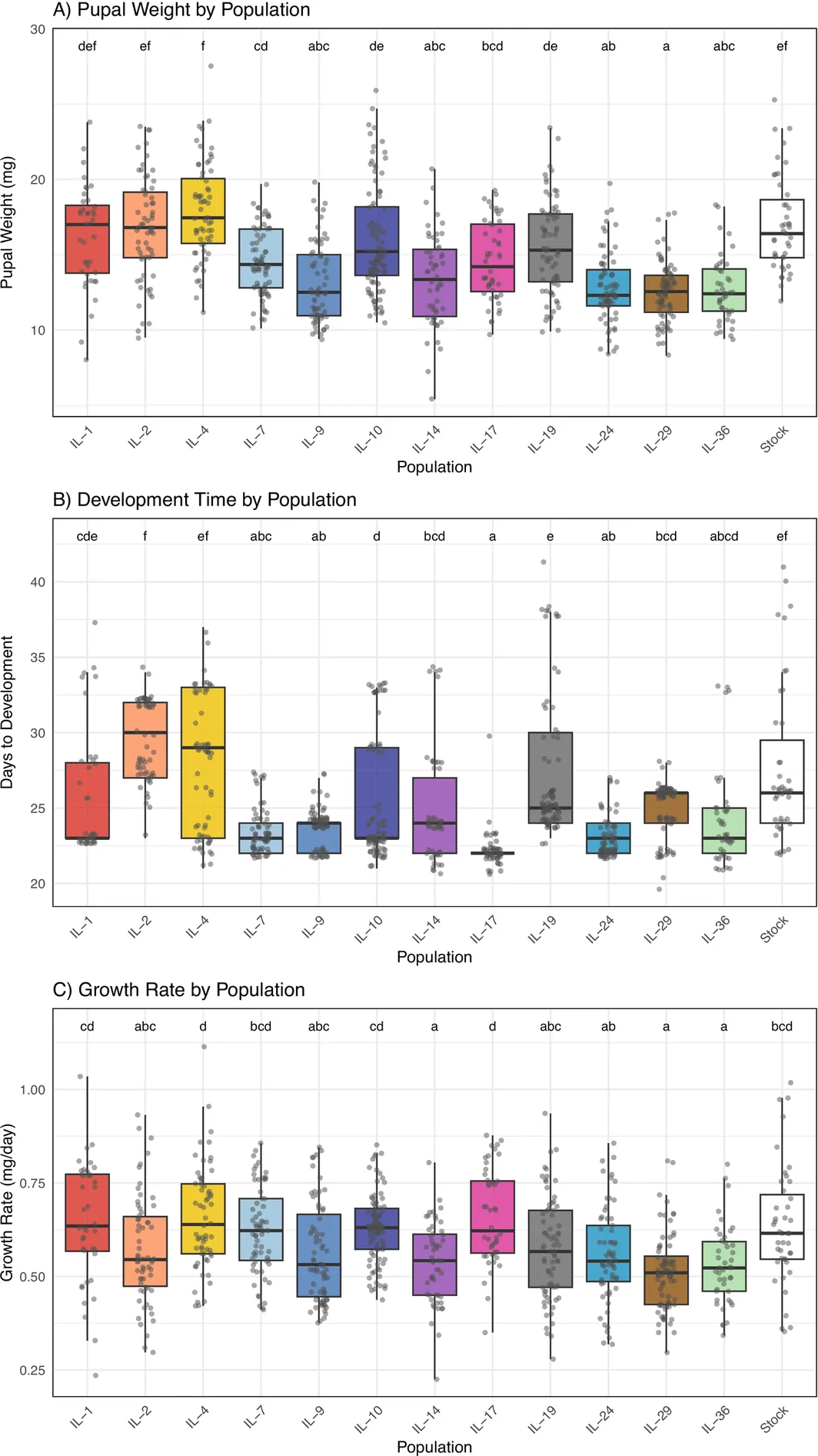
Life‐history trait variation among inbred lines and stock population of *Plodia interpunctella*. Boxplots show the distribution of (A) pupal weight (mg), (B) development time (days to pupation), and (C) growth rate (mg day^−1^) for each of the 12 inbred lines and the outbred stock population. Points represent individual measurements; boxes show the interquartile range with the median as the central line. Letters above each panel indicate statistically distinct groups based on Tukey post hoc comparisons following one‐way ANOVA (populations sharing a letter do not differ significantly at *p* < 0.05). Populations are ordered consistently across all panels.

### 3.4 Life‐History and Resistance Relationships

Despite the association between genomic structure and resistance, life‐history PC1 was not significantly correlated with LD50 (*r* = 0.23, *p* = 0.47; Figure 5). Examination of individual trait correlations confirmed this pattern: growth rate (*r* = −0.11, *p* = 0.73), pupal weight (*r* = −0.28, *p* = 0.35) and development time (*r* = −0.32, *p* = 0.29) were each non‐significantly correlated with LD50 when considered independently (Figure S8). These results indicate that position along this composite life‐history axis does not predict pathogen resistance across this set of inbred lines. To assess whether the absence of a life‐history‐resistance correlation reflects a broader dissociation between life‐history variation and genomic structure, we tested whether life‐history PC1 was itself correlated with genomic PC1 and PC2 (both from the same inbred‐only, CDS‐restricted PCA underlying Figure 1). Life‐history PC1 was not significantly correlated with genomic PC1 (*r* = −0.37, *p* = 0.235) or genomic PC2 (*r* = −0.08, *p* = 0.80), indicating that life‐history variation among these lines is not associated with either of the primary axes of genomic differentiation examined in this study, including the axis that predicts resistance.

**FIGURE 5:**
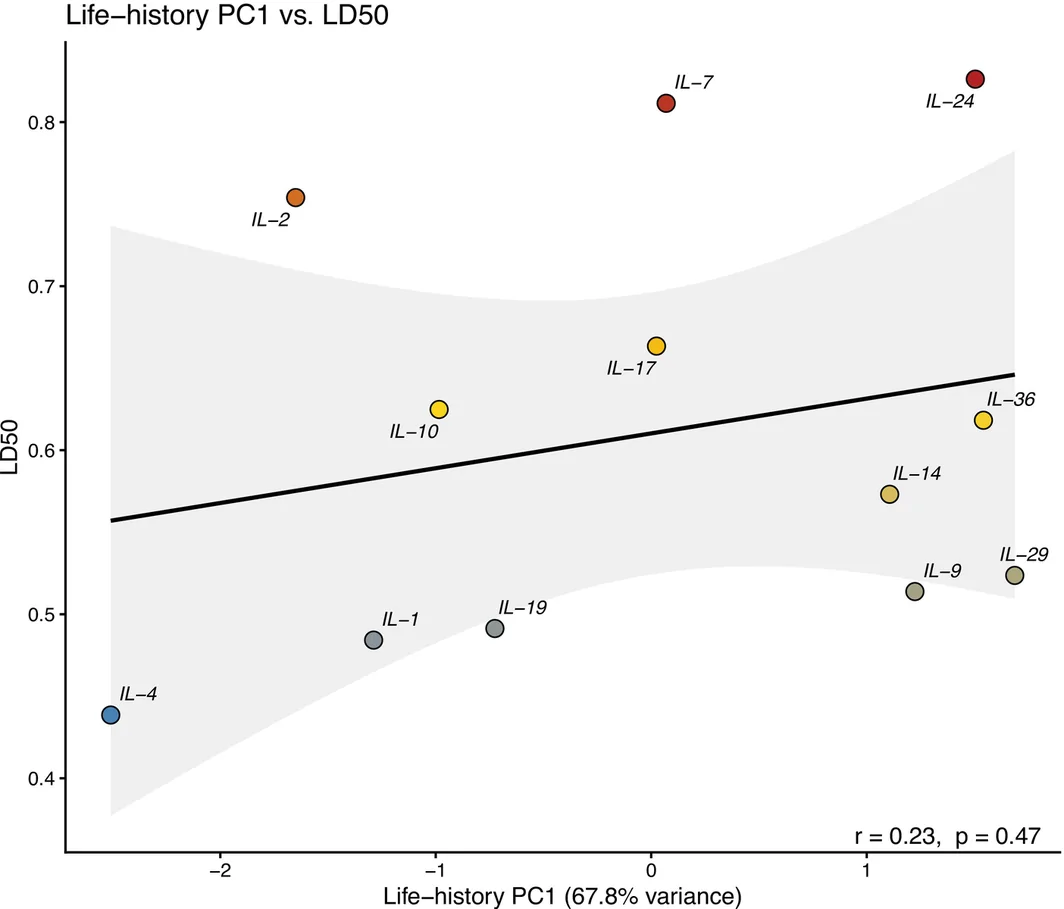
Life‐history PC1 is not significantly correlated with granulovirus resistance across inbred lines of *Plodia interpunctella*. Each point represents one of the 12 inbred lines, plotted by its score along the first principal component of the life‐history PCA (explaining 67.8% of trait variance, with all three traits loading negatively) against population‐mean LD50. Higher PC1 scores correspond to faster‐developing, lighter populations with slower growth rates. The red dashed line shows the linear regression fit. The correlation is weak and non‐significant (*r* = 0.23, *p* = 0.47), indicating that position along this composite life‐history axis does not predict resistance to PiGV across these lines. The stock population is excluded from this analysis. This contrasts with the significant negative correlation between genomic PC1 and LD50 (*r* = −0.65, *p* = 0.022; Figure 1).

To explore whether resistance‐growth relationships might nonetheless emerge at a finer scale, we used *k*‐means clustering to partition inbred lines into groups based on their resistance phenotypes (population‐mean LD50). Model selection using within‐cluster sum of squares and percentage improvement in WSS supported *k* = 3 as the optimal number of clusters (Figure S9, Table S7), corresponding to groups with high (*n* = 3), low (*n* = 5) and moderate resistance (*n* = 4; Figure S10, Table S8). Within each cluster, the relationship between growth rate and LD50 was non‐significant (Figure S11), though the low‐resistance cluster approached significance (*r* = −0.88, *p* = 0.051, *n* = 5), and all clusters had small sample sizes (*n* = 3–5). These within‐cluster analyses should therefore be considered hypothesis‐generating rather than confirmatory. We detected no consistent relationship between growth rate and resistance at either the global or within‐cluster scale.

## 4 Discussion

Our findings provide new insight into the genetic architecture of pathogen resistance in *Plodia interpunctella*. Position along genomic PC1, the primary axis of differentiation in the CDS‐restricted PCA, was significantly associated with LD50 (*r* = −0.65, *p* = 0.022), whereas the composite life‐history axis and its component traits were not. No individual PCA‐LD50 window reached formal significance after multiple‐testing correction, and the resistance‐associated signal was distributed broadly across the genome. Together, these results support a genetically structured resistance phenotype and are consistent with a diffuse, potentially polygenic basis that is not detectably coupled to the developmental life‐history variation measured here. These inferences are necessarily tempered by the modest number of inbred lines available for genomic analysis (*n* = 12). One additional experimental limitation applies to all line‐level resistance comparisons: because populations emerged asynchronously, population identity was confounded with assay day. Standardized virus aliquots and handling were used to minimize systematic day‐to‐day effects, but line and day effects cannot be formally separated. We therefore interpret the genomic PC1‐LD50 result as an association between genomic structure and the measured line‐level resistance phenotype, rather than as definitive evidence that genomic differences alone caused the observed LD50 variation.

The genomic PC1‐LD50 association reflects position along a specific axis of genomic differentiation rather than generalized genomic divergence: overall pairwise genomic distance was not significantly associated with pairwise differences in LD50. ROH coverage was also positively correlated with LD50 across inbred lines (*r* = 0.68, *p* = 0.016), suggesting that genomic PC1 and line‐level homozygosity may be partially confounded in this dataset. We therefore cannot distinguish effects associated with broad genomic structure from those arising through line‐specific differences in inbreeding or homozygosity. Drift during sibling‐pair mating could have altered the frequencies of resistance‐associated variants, although for complex traits under stabilizing selection, segregating variants are generally expected to include neutral or deleterious effects rather than being systematically resistance‐enhancing (Sella and Barton 2019). Weak indirect selection may also have occurred because the most viable of five sibling‐pair replicates, assessed on general health rather than resistance, was chosen each generation. Such selection was likely weak relative to drift, but our design cannot fully exclude a contribution from either process. Each genomic sample comprised pooled DNA from 2 to 3 individuals, such that allelic differences among individuals within a pool can appear as heterozygous calls and fragment inferred ROH tracts. In addition, the undocumented breeding history preceding the final three generations prevents reconstruction of the realized pedigree inbreeding coefficient of each line. For these reasons, we use ROH primarily as a descriptive measure of long homozygous sequence and do not interpret the positive ROH‐LD50 association as evidence that inbreeding itself increases resistance. Disentangling the contributions of genomic structure, homozygosity and locus‐specific effects will require individual‐level sequencing and a larger panel of lines spanning well‐documented inbreeding histories. A larger panel of lines would also allow heritability of resistance to be estimated with meaningful precision, which the present sample size (*n* = 12) does not support.

Our analyses provide little support for attributing resistance variation to a small number of identifiable major‐effect loci. No PCA‐LD50 window survives genome‐wide multiple‐testing correction; resistance‐associated signal was distributed broadly across windows, and the 10 convergent regions showed no enrichment for genes with known immune function relative to randomly drawn windows. We therefore do not nominate candidate genes. Instead, the distributed pattern is consistent with a diffuse, potentially polygenic basis involving many genomic regions of individually small effect. Given that these associations rest on 12 phenotypic observations, we cannot cleanly distinguish such an architecture from limited power to resolve stronger loci, and we regard the convergent regions as provisional. They are reported (Table S5) to document where signal is concentrated relative to the genomic background, not as validated resistance loci. This interpretation is also broadly consistent with quantitative‐genetic work in this system (Roberts et al. 2020), which found resistance‐associated genetic variance distributed across multiple loci rather than concentrated in a single major‐effect factor.

If confirmed, a diffuse, polygenic basis would also have consequences for how resistance in this system is expected to evolve. Simple gene‐for‐gene models of host–parasite coevolution assume that resistance and infectivity are each governed by a small number of interacting loci, allowing coevolution to proceed through allele frequency changes at individual genes (reviewed in Bolnick et al. 2025). An architecture distributed across many loci is instead more compatible with quantitative frameworks in which coevolution shifts the distribution of resistance phenotypes rather than turning over a small number of discrete resistance alleles.

A diffuse architecture also bears on the absence of a resistance‐life‐history correlation, although the present data do not resolve locus‐level pleiotropy. Nevertheless, life‐history PC1 was not correlated with genomic PC1, the axis associated with resistance (*r* = −0.371, *p* = 0.235), or genomic PC2 (*r* = −0.08, *p* = 0.80). Resistance and life‐history variation therefore do not appear to track the same major axes of genomic differentiation among these lines. The conclusion concerns broad patterns of genomic covariance; whether any specific gene contributes to both traits is not addressed by this analysis.

Two further limitations constrain gene‐level inference in this system. Genome annotation for *P. interpunctella* remains incomplete, and many genes falling within the convergent regions are uncharacterized or annotated only by homology to better‐studied insects, particularly *Drosophila*. Separately, at a window size of 250 kb each window contains many genes, so even a window with strong support would not by itself isolate a causal locus. Fine‐mapping resistance in this system will require a substantially larger panel of lines.

Resistance in this system is also likely to depend on mechanisms that window‐based association analyses are not well suited to detect. The peritrophic matrix represents an important first line of defence against PiGV, as the virus must cross this barrier to establish infection in gut epithelial cells (Begon et al. 1993), and variation in peritrophic matrix integrity across inbred lines may contribute to resistance differences. RNAi is a critical antiviral defence mechanism in insects, and lines may likewise vary in RNAi efficiency (Gammon and Mello 2015; Lebenzon and Toxopeus 2024). Together with the diffuse genomic signal we observe, these considerations suggest that multiple pathways could contribute to resistance variation in this system.

The contemporary outbred stock population was genomically distinct from the inbred lines, separating clearly on PC1 of the global PCA and forming two clusters consistent with its dual‐source origin (Berkeley and Oakland). As expected for an independently assembled, outbred reference population, the stock pools collectively harboured substantially more genomic variation than any individual inbred line. The stock's LD50 (0.5185) falls within the lower range of inbred‐line values, providing a useful phenotypic reference showing that the resistance range among the inbred lines overlaps with and extends beyond that observed in this independently sampled outbred population. Importantly, this contemporary stock is not the ancestral laboratory population from which the inbred lines were derived. It therefore cannot be used to infer allele frequencies, resistance distributions or standing genetic variation in that original founder population. The approximately two‐fold range in LD50 among the inbred lines nonetheless demonstrates substantial resistance variation across the genetic backgrounds represented in this panel.

Despite the clear composite life‐history axis captured by PC1, we find no significant correlation between life‐history position and resistance. The absence of a significant relationship between resistance and life‐history traits might superficially suggest the existence of genotypes capable of maximizing all fitness components simultaneously, so‐called ‘Darwinian demons’ (Law 1979). However, this interpretation is almost certainly premature: growing evidence indicates that resistance‐life‐history trade‐offs in *Plodia* are context‐dependent and may not be detectable across all experimental designs. Bartlett et al. (2020) found that directly selecting on development time did not produce a corresponding change in resistance, even though selection on resistance itself had previously revealed the trade‐off (Boots 2011; Boots and Begon 1993). This asymmetry suggests that the relationship between resistance and development time is not a simple, invariant genetic constraint and may depend on the direction of selection. Visher et al. (2022) demonstrated that trade‐offs emerge under low nutrition conditions but not consistently across environments, confirming that their detectability depends on ecological context. This decoupling extends to genomic structure itself: life‐history PC1 is not significantly associated with either of the primary axes of genomic differentiation examined in this study (genomic PC1 or PC2), indicating that life‐history variation is not simply organized along the same genomic architecture that predicts resistance. We also considered eclosion rate as an additional life‐history axis distinct from the developmental traits captured by PC1; it did not differ significantly among lines or correlate with resistance. Fecundity remains a notable gap: despite repeated attempts, it has proven technically difficult to measure reliably in this system, and its relationship to resistance and to the developmental traits examined here remains an open question for future work.

Previous studies demonstrated a genotypic trade‐off between resistance and growth rate using inbred lines derived from the same ancestral laboratory stock population (Bartlett et al. 2018) and confirmed this trade‐off through selection experiments (Boots 2011; Boots and Begon 1993), making it one of the few evolutionary trade‐offs confirmed by multiple independent experimental approaches. Our results do not replicate this trade‐off across life‐history traits but support a genetic contribution to resistance variation among inbred lines, extending those earlier findings by characterizing the genomic architecture of resistance and identifying regions where association signal is concentrated. This suggests that resistance has a detectable genomic component, whereas its expression as a life‐history trade‐off may be more context‐dependent than previously appreciated.

The lines examined by Bartlett et al. (2018) are distinct from those used here, although both panels derive from the same ancestral laboratory stock. The contrast therefore suggests that detectability of the resistance‐growth trade‐off may depend on the genetic backgrounds represented in a given panel and their subsequent breeding histories, rather than reflecting change within the same lines over time.

Resource availability may nonetheless shape whether trade‐offs are expressed. Our lines were maintained under identical, resource‐rich laboratory conditions, and resource allocation theory predicts costs of immunity are most readily detected when resources are genuinely limiting (Lochmiller and Deerenberg 2000; Sheldon and Verhulst 1996), with experimental work in this system supporting such resource dependence (Roberts et al. 2020; Visher et al. 2022). We note, however, that Bartlett et al. (2018) detected a resistance‐growth trade‐off under comparable dietary conditions, so resource availability alone cannot account for the difference between their results and ours. Whether trade‐offs would emerge among our lines under resource limitation remains an open question.

The persistence of resistance variation also does not require a fixed resistance‐life‐history trade‐off under all conditions. Classical models predict that costs of resistance can maintain resistance polymorphism when the relative fitness of resistant and susceptible genotypes varies with pathogen pressure (Boots and Bowers 1999; Boots and Haraguchi 1999). Empirical work in *Plodia* further shows that nutritional environment, historical contingency and the direction of selection influence whether resistance costs are expressed (Bartlett et al. 2020; Roberts et al. 2020; Visher et al. 2022). Such context dependence provides multiple routes by which resistance variation could persist under spatially or temporally heterogeneous selection even when a trade‐off is not detectable under every experimental condition. The present panel cannot determine which, if any, of these processes maintained variation in the ancestral laboratory stock, whose genetic composition and evolutionary history are incompletely documented.

Our results reinforce the value of examining resistance variation at the genomic level rather than relying solely on phenotypic correlations among traits. The genomic PC1‐LD50 association, combined with the absence of detectable associations between LD50 and the measured life‐history traits, indicates that resistance covaries more strongly with a major axis of genomic differentiation than with developmental life‐history variation under the conditions examined here. This association is nonetheless sensitive to the inclusion of IL‐4, an extreme line on genomic PC1 and the most susceptible line (excluding it gives *r* = −0.60, *p* = 0.052), and to the choice of PCA standardization (an allele‐frequency‐standardized analysis gives *r* = −0.58, *p* = 0.049). Both underscore the value of increasing the number of inbred lines in future work. The approximately two‐fold variation in LD50 demonstrates substantial resistance diversity across the genetic backgrounds sampled here. Because the contemporary outbred stock was assembled independently, its genomic distinctiveness should be interpreted as reference context only rather than as evidence about the allele frequencies or resistance distribution of the ancestral laboratory stock.

Although all 12 inbred lines descend from the same ancestral laboratory stock, we cannot determine how much of their present‐day genomic differentiation was already present as structure within that founder population versus accumulated subsequently during independent line propagation. The geographic origin and genetic composition of the ancestral stock are not documented, and the stock itself is no longer available for genomic comparison. Moreover, the contemporary Oakland/Berkeley stock was assembled independently and therefore cannot be used to reconstruct variation in the ancestral population. We consequently interpret genomic PC1 as an axis of present‐day differentiation among the inbred lines without attributing its origin specifically to drift, inbreeding or pre‐existing population structure.

This study expands upon existing research in this system (Bartlett et al. 2018, 2020; Boots 2011; Boots and Begon 1993) by providing the first genome‐wide characterization of resistance variation in *P. interpunctella*. Future work examining how these inbred line genotypes perform under variable ecological conditions, including resource limitation, temperature fluctuation and competitive pressures, would help determine whether life‐history trade‐offs with resistance emerge under conditions more representative of the natural environment. Future genomic work in this system would also benefit from individual‐level sequencing rather than pooled extractions, which would eliminate the within‐individual/between‐individual heterozygosity ambiguity discussed above, and from additional generations of documented sibling‐pair mating to more fully separate the inbreeding history of individual lines from that of their shared source population.

## Author Contributions

P. Signe White: conceptualization, data collection, formal analysis, visualization, writing – original draft, writing – review and editing. Johnathan Lo: genomic data processing and bioinformatics. Jonathan Barajas, Hailey Burnsed, Noah Parker, Zucel Rodriguez, and Natalie Walzer: data collection. Peter H. Sudmant: resources, supervision. Mike Boots: conceptualization, funding acquisition, supervision, writing – review and editing.

## Funding

This work is funded by an National Science Foundation (NSF) DEB EEID grant (award no. 2011109) to M.B.

## Disclosure

Benefits Generated: Benefits from this research accrue from the sharing of our data and results on public databases as described above. All biological samples used in this study were derived from laboratory‐maintained *Plodia interpunctella* colonies at the University of California, Berkeley. The contemporary outbred stock was originally established from household infestations in the Berkeley/Oakland area; no international collection or sampling of genetic resources from provider countries was conducted, and the Nagoya Protocol is not applicable to this study.

## Ethics Statement

This study used invertebrate insects (*Plodia interpunctella*) and does not require institutional ethics approval under applicable regulations.

## Conflicts of Interest

The authors declare no conflicts of interest.

## Supporting information


**Table S1:** Larval diet composition. Diet used for all *P. interpunctella* population maintenance and bioassays.
**Table S2:** Present‐study generation at time of phenotypic assessment. Generations at which phenotypic assays were conducted for each inbred line. ‘Generation’ refers to the generation count used during the present study, beginning when strict sibling‐pair mating was resumed (G1), rather than the total number of generations since the line was originally established. G1–G3 comprised the three documented generations of strict full‐sibling mating; at G4, lines were scaled to replicate pots and subsequently propagated under the standardized maintenance protocol described in the main Methods. Thus, G5 denotes the fifth present‐study generation, not five consecutive generations of full‐sibling mating. These generation numbers indicate the stage of present‐study propagation at phenotypic assessment and genomic sampling and should not be interpreted as total pedigree inbreeding depth.
**Figure S1:** Correlation between mean pupal weight and mean development time across *Plodia interpunctella* populations. Each point represents the population mean for one of the 12 inbred lines or the outbred stock population. Error bars show ±1 standard error of the mean in both dimensions. The solid line shows the linear regression fit across all 13 populations and the shaded ribbon indicates the 95% confidence interval. The positive correlation (*r* = 0.74, *p* = 0.004) indicates that heavier pupae tend to develop more slowly, consistent with coordinated variation in developmental traits among populations.
**Figure S2:** Neighbour‐joining trees of genomic relatedness among inbred lines of *Plodia interpunctella* are consistent between CDS‐restricted and genome‐wide variant sets. Trees were constructed from pairwise genetic distances (Euclidean distance on genotype calls) calculated across (A) CDS‐restricted variants (270,375 variants) and (B) genome‐wide, complete‐call‐filtered variants (1,717,334 variants). Tip labels are coloured by LD50 resistance category: blue = susceptible (low LD50), yellow/gold = intermediate, red = resistant (high LD50). Scale bar represents 50 units of Euclidean genetic distance. Both trees identify IL‐17 and IL‐36 as the most closely related pair and IL‐4 as the most genomically divergent line. The two distance matrices are significantly concordant (Mantel test, *r* = 0.57, *p* < 0.001, 9999 permutations). Overall genomic distance, as depicted here, was not significantly associated with pairwise differences in LD50 under either variant set (CDS‐restricted: Mantel *r* = 0.16, *p* = 0.14; genome‐wide: *r* = 0.10, *p* = 0.27); resistance is instead associated with genomic PC1, the primary axis of genomic differentiation (Figure 3, Figure S13).
**Figure S3:** Runs of homozygosity show elevated homozygosity in *Plodia interpunctella* lines. Runs of homozygosity were identified from whole‐genome sequencing data using bcftools roh v1.16 with a default allele frequency of 0.4. (A) Percentage of the genome contained within ROH for each of the 12 inbred lines. Bar colour indicates LD50 (blue = susceptible, red = resistant). The dashed line indicates the mean across all inbred lines (26.1%). ROH coverage was positively correlated with LD50 across inbred lines (*r* = 0.68, *p* = 0.016). (B) Distribution of genome‐wide ROH coverage for the stock population (*n* = 84 pooled samples) and the 12 inbred lines (*n* = 12). ROH coverage was substantially higher in the inbred lines than in stock pools overall (26.1% vs. 7.3%). Because both inbred‐line and stock sequencing libraries were generated from pools of 2–3 individuals, these ROH values are best interpreted as descriptive measures of long homozygous sequences; pooling is expected to be especially consequential in the genetically heterogeneous stock pools but may also affect inbred‐line estimates where residual within‐line variation remains.
**Figure S4:** Dose–response curves for granulovirus infection across all *Plodia interpunctella* populations. Infection proportion is plotted against log_10_(relative PiGV concentration) for the 12 inbred lines and the stock population, shown together in a single overlaid plot. Solid lines show predicted infection probabilities from a binomial logistic GLM fitted with dose × population interaction terms; shaded ribbons indicate 95% confidence intervals. Points represent observed infection proportions (number infected/total exposed) at each dose. Populations are coloured as indicated in the legend; stock is shown in black.
**Figure S5:** Observed and predicted infection proportions by population, shown in separate panels for each of the 12 inbred lines and the stock population (compare Figure S4, which overlays all populations on a single plot). Infection proportion is plotted against log_10_(relative PiGV concentration) for each of the 12 inbred lines and the stock population of *Plodia interpunctella*. Solid lines show predicted infection probabilities from a binomial logistic GLM fitted with dose × population interaction terms; shaded ribbons indicate 95% confidence intervals. Red dashed lines and points show observed infection proportions (number infected/total exposed) at each dose.
**Figure S6:** Log‐odds of infection as a function of relative PiGV concentration for each *Plodia interpunctella* population. Log‐odds of infection (linear predictor from the binomial logistic GLM) are plotted against log_10_(relative PiGV concentration) for the 12 inbred lines and the stock population. Differences in both slope and intercept reflect heterogeneity in dose–response relationships among populations, consistent with the significant dose × population interaction in the logistic GLM (Table S3). Populations are coloured as indicated in the legend.
**Table S3:** Binomial Logistic GLM—Analysis of Deviance. Model: binomial, link: logit. Response: cbind(Infection_Status, Total_Exposed − Infection_Status).
**Table S4:** LD50 and eclosion success by population. LD50 and SE were calculated from binomial infection assay data using a logistic GLM and the MASS::dose.p function. LD50 values are expressed as relative stock concentration, with the undiluted common working PiGV stock defined as 1×. Eclosion rate is the proportion of individuals that successfully eclosed among those that pupated during the life‐history assays. Eclosion rate did not differ significantly among inbred lines (*χ* ^2^ = 11.32, df = 11, *p* = 0.42) and was not significantly correlated with LD50 across lines (*r* = −0.49, *p* = 0.11).
**Table S5:** Convergent genomic windows ranking highly by both PVE and WZA. Ten contiguous regions across five chromosomes met the pre‐specified joint criterion (top 1%/2% by both PVE and WZA) comprising 21 jointly high‐ranking windows. Peak window is the highest‐PVE window within each region. Sign of the window‐level PCA correlation is arbitrary and not reported; PVE (*r* ^2^) is a sign‐invariant measure of window‐level association strength. The joint top‐ranking criterion is a descriptive ranking rule rather than a formal significance test.
**Figure S7:** Quantile‐quantile (QQ) plots for genome‐wide window‐based analyses of granulovirus resistance in *Plodia interpunctella*. The red dashed line indicates the expectation under the null (slope = 1). (Left) PCA‐LD50 approach: observed −log_10_(*p*) values are plotted against expected values under the null hypothesis of no association. Observed values deviate above the null diagonal across much of the distribution, while the most extreme points converge back toward the expected line rather than diverging further. (Right) WZA approach: observed |Z_W,norm| values are plotted against expected values under a half‐normal null distribution. Because SNPs within windows remain linked, WZA is treated as a ranking statistic rather than a formal window‐level significance test; this panel is therefore used as a diagnostic of the normalized score distribution. Most windows follow the half‐normal expectation, with a distinct upper‐tail subset corresponding to windows prioritized by the joint PVE/WZA ranking criterion (Table S5). These QQ plots are diagnostic and do not themselves establish formal significance.
**Table S6:** Immune gene enrichment permutation test in convergent windows. For each of two keyword sets defined for the enrichment analysis, the peak window within each of the 10 convergent regions (Table S5) was intersected with the *P. interpunctella* genome annotation (172 genes total). Observed keyword‐match counts were compared against a null distribution generated by drawing 10 windows at random from the full set of 2164 retained windows, 10,000 times. Neither keyword set showed enrichment; the strict‐list observed count was below the null expectation.
**Figure S8:** LD50 is not significantly correlated with individual life‐history traits across inbred lines of *Plodia interpunctella*. Each point represents one of the 12 inbred lines. Population‐mean LD50 is plotted against (A) mean growth rate (mg day^−1^), (B) mean pupal weight (mg) and (C) mean development time (days to pupation). Dashed lines show linear regression fits. None of the correlations are statistically significant: growth rate (*r* = −0.11, *p* = 0.73), pupal weight (*r* = −0.28, *p* = 0.35) and development time (*r* = −0.32, *p* = 0.29). The stock population is excluded from all panels. These results are consistent with the non‐significant life‐history PC1‐LD50 correlation (Figure 5) and indicate that resistance was not detectably associated with the measured life‐history traits across these inbred lines.
**Figure S9:** *K*‐means model selection for clustering of granulovirus resistance phenotypes across inbred lines of *Plodia interpunctella*. *K*‐means clustering was applied to population‐mean LD50 values for *k* = 1 to 5, with 25 random starts per *k* (seed = 42). (A) Within‐cluster sum of squares (WSS) as a function of *k*, showing a clear elbow at *k* = 3 beyond which additional clusters yield diminishing returns. (B) Percentage reduction in WSS relative to the previous *k*, showing high and stable improvement from *k* = 1 to *k* = 3 followed by a marked drop at *k* = 4. Red dashed lines indicate the selected value of *k* = 3, which is supported by both criteria. Clustering results are presented in Figures S10 and S11.
**Table S7:** *K*‐means model‐selection metrics for resistance clustering. *K* = 3 was selected based on the elbow in within‐cluster sum of squares (WSS) and the marked reduction in incremental WSS improvement beyond *k* = 3. AIC and BIC are shown for completeness but continue to decrease through *k* = 5 and therefore do not independently identify *k* = 3 as an optimum.
**Figure S10:** Distribution of LD50 values within resistance clusters identified by *k*‐means clustering of *Plodia interpunctella* inbred lines. Boxplots show the distribution of population‐mean LD50 values for each of the three clusters identified by *k*‐means clustering (*k* = 3) applied to LD50 values across the 12 inbred lines. Points represent individual inbred lines. Boxes show the interquartile range with the median as the central line. Clusters are colour‐coded by resistance level: blue = low resistance (*n* = 5 lines), gold = moderate resistance (*n* = 4 lines), red = high resistance (*n* = 3 lines). Cluster assignments were used to examine within‐cluster relationships between resistance and life‐history traits (Figure S11).
**Table S8:** Cluster assignments (*k* = 3).
**Figure S11:** Relationship between growth rate and granulovirus resistance within resistance clusters of *Plodia interpunctella* inbred lines. Each point represents one inbred line, plotted by mean growth rate (mg day^−1^) against population‐mean LD50, with panels separated by resistance cluster assignment (see Figure S10). Lines show within‐cluster linear regression fits. Pearson correlation coefficients and *p*‐values are shown for each cluster. No within‐cluster correlation is statistically significant at *p* < 0.05, though the low‐resistance cluster approaches significance (*r* = −0.88, *p* = 0.051, *n* = 5), with higher growth rate associated with lower resistance. All within‐cluster sample sizes are small (*n* = 3–5) and these analyses should be considered hypothesis‐generating rather than confirmatory. Axes are standardized across panels to facilitate visual comparison among clusters.
**Figure S12:** Window‐level resistance‐associated signal across genomic windows in *Plodia interpunctella* is more diffuse than expected under LD50 permutation. Windows were ranked by PVE (*r* ^2^) and cumulative share of summed window‐level PVE was plotted against the fraction of windows included. These percentages summarize the distribution of window‐level *r* ^2^ values and are not an additive decomposition of phenotypic variance. The blue line shows the observed distribution across the 2164 retained windows; the grey band shows the 95% null envelope generated by permuting LD50 across 12 lines 1000 times and recomputing the PVE distribution under each permutation (dashed line: null mean). The observed cumulative PVE curve never exceeds the 95% null envelope at any rank and falls below its lower bound beyond approximately 24% of windows, indicating that window‐level association strength is less concentrated among the highest‐ranking windows and more broadly distributed across the genome than in the permuted datasets.
**Figure S13:** Genomic PC1‐LD50 association is similar under CDS‐restricted and genome‐wide variant sets. Each point represents one of the 12 inbred lines, plotted by its score along the first principal component of genomic PCA against population‐mean LD50, coloured by LD50 (blue = susceptible, gold = intermediate, red = resistant). (A) CDS‐restricted PCA (270,375 variants; *r* = −0.65, *p* = 0.022). (B) Genome‐wide, complete‐call‐filtered PCA (1,717,334 variants; *r* = −0.57, *p* = 0.052). The solid line shows the linear regression fit with 95% confidence interval. The association is similar in direction and magnitude between the two variant sets, though the genome‐wide analysis narrowly misses conventional significance, which may reflect dilution of the resistance‐associated signal by a larger proportion of neutral genome‐wide variation.

## Data Availability

All analysis code is available at https://github.com/sigwhite1/genomics‐resistance. Processed data files and a snapshot of the code repository are deposited in Dryad at https://doi.org/10.5061/dryad.z08kprrvz. Raw genomic sequencing data (FASTQ files) for all 12 inbred lines and 84 pooled outbred stock samples are available from the NCBI Sequence Read Archive (BioProject PRJNA1461375; SRA Study SRP697324).
