# Supplementary material for: Genomic Structure Predicts Granulovirus Resistance Without a Detectable Life‐History Trade‐Off in Plodia interpunctella
Source: Mol Ecol. 2026 Sep 28;35(19):e70563. doi: 10.1111/mec.70563 (PMC13619880; doi:10.1111/mec.70563)
Supplement: Supplementary file 1 [file MEC-35-e70563-s001.docx]

## **Table S1. Larval diet composition**

Diet used for all *P. interpunctella* population maintenance and bioassays.

| **Ingredient** | **Brand / Cat. No.** | **Amount** |
| --- | --- | --- |
| Cereal mix (see below) |  | 500 g |
| Brewer's yeast | MP Biomedicals,  Cat. No. 0290331205 | 100 g |
| Methyl paraben (methyl-p-hydroxybenzoate) | MP Biomedicals,  Cat. No. MP21023415 | 2.2 g |
| Sorbic acid | Spectrum Chemical,  Cat. No. S1517 | 2.2 g |
| Glycerol | Macron Fine Chemicals,  Cat. No. 56-81-5 | 125 mL |
| Honey | Dutch Gold,  SKU: 73138-30560-6 | 125 mL |

**Cereal Mix Recipe:**

| **Ingredient** | **Brand** | **Amount** |
| --- | --- | --- |
| Oatmeal cereal | Earth's Best | 681 g |
| Wheat bran | Bob's Red Mill | 454 g |
| Rice flour | Bob's Red Mill | 365 g |

## **Table S2. Present-study generation at time of phenotypic assessment**

Generations at which phenotypic assays were conducted for each inbred line. "Generation" refers to the generation count used during the present study, beginning when strict sibling-pair mating was resumed (G1), rather than the total number of generations since the line was originally established. G1-G3 comprised the three documented generations of strict full-sibling mating; at G4, lines were scaled to replicate pots and subsequently propagated under the standardized maintenance protocol described in the main Methods. Thus, G5 denotes the fifth present-study generation, not five consecutive generations of full-sibling mating. These generation numbers indicate the stage of present-study propagation at phenotypic assessment and genomic sampling and should not be interpreted as total pedigree inbreeding depth.

| **Population** | **Generation** |
| --- | --- |
| IL-1 | 7 |
| IL-2 | 8 |
| IL-4 | 8 |
| IL-7 | 7 |
| IL-9 | 10 |
| IL-10 | 9 |
| IL-14 | 13 |
| IL-17 | 5 |
| IL-19 | 7 |
| IL-24 | 5 |
| IL-29 | 8 |
| IL-36 | 5 |

**Figure S1. Correlation between mean pupal weight and mean development time across *Plodia interpunctella* populations.** Each point represents the population mean for one of the 12 inbred lines or the outbred stock population. Error bars show ±1 standard error of the mean in both dimensions. The solid line shows the linear regression fit across all 13 populations and the shaded ribbon indicates the 95% confidence interval. The positive correlation (r = 0.74, p = 0.004) indicates that heavier pupae tend to develop more slowly, consistent with coordinated variation in developmental traits among populations.

**Figure S2. Neighbor-joining trees of genomic relatedness among inbred lines of *Plodia interpunctella* are consistent between CDS-restricted and genome-wide variant sets.** Trees were constructed from pairwise genetic distances (Euclidean distance on genotype calls) calculated across **(A)** CDS-restricted variants (270,375 variants) and **(B)** genome-wide, complete-call-filtered variants (1,717,334 variants). Tip labels are colored by LD50 resistance category: blue = susceptible (low LD50), yellow/gold = intermediate, red = resistant (high LD50). Scale bar represents 50 units of Euclidean genetic distance. Both trees identify IL-17 and IL-36 as the most closely related pair and IL-4 as the most genomically divergent line. The two distance matrices are significantly concordant (Mantel test, r = 0.57, p < 0.001, 9,999 permutations). Overall genomic distance, as depicted here, was not significantly associated with pairwise differences in LD50 under either variant set (CDS-restricted: Mantel r = 0.16, p = 0.14; genome-wide: r = 0.10, p = 0.27); resistance is instead associated with genomic PC1, the primary axis of genomic differentiation (Figure 3, Figure S13).

**Figure S3. Runs of homozygosity show elevated homozygosity in *Plodia interpunctella* lines**. Runs of homozygosity were identified from whole-genome sequencing data using bcftools roh v1.16 with a default allele frequency of 0.4. (A) Percentage of the genome contained within ROH for each of the 12 inbred lines. Bar color indicates LD50 (blue = susceptible, red = resistant). The dashed line indicates the mean across all inbred lines (26.1%). ROH coverage was positively correlated with LD50 across inbred lines (r = 0.68, p = 0.016). (B) Distribution of genome-wide ROH coverage for the stock population (n = 84 pooled samples) and the 12 inbred lines (n = 12). ROH coverage was substantially higher in the inbred lines than in stock pools overall (26.1% vs. 7.3%). Because both inbred-line and stock sequencing libraries were generated from pools of 2-3 individuals, these ROH values are best interpreted as descriptive measures of long homozygous sequences; pooling is expected to be especially consequential in the genetically heterogeneous stock pools but may also affect inbred-line estimates where residual within-line variation remains.

Log10(relative PiGV concentration)

**Figure S4. Dose-response curves for granulovirus infection across all *Plodia interpunctella* populations**. Infection proportion is plotted against log10(relative PiGV concentration) for the 12 inbred lines and the stock population, shown together in a single overlaid plot. Solid lines show predicted infection probabilities from a binomial logistic GLM fitted with dose × population interaction terms; shaded ribbons indicate 95% confidence intervals. Points represent observed infection proportions (number infected / total exposed) at each dose. Populations are colored as indicated in the legend; stock is shown in black.

Log10(relative PiGV concentration)

**Figure S5. Observed and predicted infection proportions by population, shown in separate panels for each of the 12 inbred lines and the stock population** (compare Figure S4, which overlays all populations on a single plot). Infection proportion is plotted against log10(relative PiGV concentration) for each of the 12 inbred lines and the stock population of *Plodia interpunctella*. Solid lines show predicted infection probabilities from a binomial logistic GLM fitted with dose × population interaction terms; shaded ribbons indicate 95% confidence intervals. Red dashed lines and points show observed infection proportions (number infected/total exposed) at each dose.

Log10(relative PiGV concentration)

**Figure S6. Log-odds of infection as a function of relative PiGV concentration for each *Plodia interpunctella* population.** Log-odds of infection (linear predictor from the binomial logistic GLM) are plotted against log10(relative PiGV concentration) for the 12 inbred lines and the stock population. Differences in both slope and intercept reflect heterogeneity in dose-response relationships among populations, consistent with the significant dose × population interaction in the logistic GLM (Table S3). Populations are colored as indicated in the legend.

**Table S3. Binomial Logistic GLM — Analysis of Deviance**

Model: binomial, link: logit. Response: cbind(Infection_Status, Total_Exposed − Infection_Status).

| **Term** | **Df** | **Deviance** | **Resid. Df** | **Resid. Dev** | **p (Chi-sq)** |
| --- | --- | --- | --- | --- | --- |
| NULL |  |  | 51 | 1480.85 |  |
| Log_Dose | 1 | 1267.24 | 50 | 213.61 | p < 0.001 *** |
| Population | 12 | 40.28 | 38 | 173.34 | p < 0.001 *** |
| Log_Dose:Population | 12 | 23.12 | 26 | 150.22 | 0.027 * |

Log_Dose [log10(relative PiGV concentration)] is the dominant predictor of infection probability. Population identity also contributes significantly, and the significant dose × population interaction indicates heterogeneity in dose-response relationships among populations, supporting population-specific LD50 estimation.

** p < 0.05; ** p < 0.01; *** p < 0.001*

**Table S4. LD50 and eclosion success by population.** LD50 and SE were calculated from binomial infection assay data using a logistic GLM and the MASS::dose.p function. LD50 values are expressed as relative stock concentration, with the undiluted common working PiGV stock defined as 1×. Eclosion rate is the proportion of individuals that successfully eclosed among those that pupated during the life-history assays. Eclosion rate did not differ significantly among inbred lines (χ² = 11.32, df = 11, p = 0.42) and was not significantly correlated with LD50 across lines (r = -0.49, p = 0.11).

| **Population** | **LD50 (relative stock concentration)** | **SE** | **Pupated (n)** | **Eclosed (n)** | **Eclosion Rate** |
| --- | --- | --- | --- | --- | --- |
| IL-1 | 0.484 | 0.057 | 64 | 50 | 0.781 |
| IL-2 | 0.754 | 0.118 | 28 | 17 | 0.607 |
| IL-4 | 0.439 | 0.065 | 47 | 37 | 0.787 |
| IL-7 | 0.811 | 0.137 | 81 | 66 | 0.815 |
| IL-9 | 0.514 | 0.075 | 84 | 71 | 0.845 |
| IL-10 | 0.625 | 0.077 | 87 | 65 | 0.747 |
| IL-14 | 0.573 | 0.047 | 89 | 67 | 0.753 |
| IL-17 | 0.663 | 0.067 | 93 | 68 | 0.731 |
| IL-19 | 0.491 | 0.068 | 43 | 35 | 0.814 |
| IL-24 | 0.826 | 0.106 | 90 | 66 | 0.733 |
| IL-29 | 0.524 | 0.063 | 78 | 63 | 0.808 |
| IL-36 | 0.618 | 0.082 | 56 | 45 | 0.804 |
| Stock | 0.518 | 0.062 | 61 | 53 | 0.869 |

**Table S5. Convergent genomic windows ranking highly by both PVE and WZA**. Ten contiguous regions across five chromosomes met the pre-specified joint criterion (top 1%/2% by both PVE and WZA) comprising 21 jointly high-ranking windows. Peak window is the highest-PVE window within each region. Sign of the window-level PCA correlation is arbitrary and not reported; PVE (r^2^) is a sign-invariant measure of window-level association strength. The joint top-ranking criterion is a descriptive ranking rule rather than a formal significance test.

| **Region** | **Chromosome** | **Region span (Mb)** | **Size (kb)** | **# windows** | **Peak window (Mb)** | **\|r\|** | **PVE** | **PVE rank** | **WZA rank** |
| --- | --- | --- | --- | --- | --- | --- | --- | --- | --- |
| 1 | NC_071295.1 | 6.625–7.125 | 500 | 3 | 6.750–7.000 | 0.85 | 0.723 | 1 | 14 |
| 2 | NC_071295.1 | 5.750–6.375 | 625 | 4 | 6.000–6.250 | 0.831 | 0.691 | 2 | 1 |
| 3 | NC_071295.1 | 7.500–7.875 | 375 | 2 | 7.625–7.875 | 0.83 | 0.689 | 3 | 23 |
| 4 | NC_071315.1 | 4.000–4.375 | 375 | 2 | 4.125–4.375 | 0.81 | 0.656 | 6 | 31 |
| 5 | NC_071315.1 | 5.250–5.500 | 250 | 1 | 5.250–5.500 | 0.806 | 0.649 | 8 | 33 |
| 6 | NC_071306.1 | 7.000–7.625 | 625 | 4 | 7.375–7.625 | 0.794 | 0.63 | 11 | 4 |
| 7 | NC_071298.1 | 7.625–7.875 | 250 | 1 | 7.625–7.875 | 0.731 | 0.534 | 24 | 41 |
| 8 | NC_071306.1 | 6.125–6.375 | 250 | 1 | 6.125–6.375 | 0.73 | 0.533 | 25 | 24 |
| 9 | NC_071298.1 | 8.250–8.500 | 250 | 1 | 8.250–8.500 | 0.717 | 0.515 | 27 | 36 |
| 10 | NC_071312.1 | 4.000–4.375 | 375 | 2 | 4.000–4.250 | 0.683 | 0.467 | 43 | 13 |

**Figure S7. Quantile-quantile (QQ) plots for genome-wide window-based analyses of granulovirus resistance in *Plodia interpunctella*.** The red dashed line indicates the expectation under the null (slope = 1). (Left) PCA-LD50 approach: observed -log_10_(p) values are plotted against expected values under the null hypothesis of no association. Observed values deviate above the null diagonal across much of the distribution, while the most extreme points converge back toward the expected line rather than diverging further. (Right) WZA approach: observed |Z_W,norm| values are plotted against expected values under a half-normal null distribution. Because SNPs within windows remain linked, WZA is treated as a ranking statistic rather than a formal window-level significance test; this panel is therefore used as a diagnostic of the normalized score distribution. Most windows follow the half-normal expectation, with a distinct upper-tail subset corresponding to windows prioritized by the joint PVE/WZA ranking criterion (Table S5). These QQ plots are diagnostic and do not themselves establish formal significance.

**Table S6. Immune gene enrichment permutation test in convergent windows.** For each of two keyword sets defined for the enrichment analysis, the peak window within each of the ten convergent regions (Table S5) was intersected with the *P. interpunctella* genome annotation (172 genes total). Observed keyword-match counts were compared against a null distribution generated by drawing ten windows at random from the full set of 2,164 retained windows, 10,000 times. Neither keyword set showed enrichment; the strict-list observed count was below the null expectation.

| **Keyword set** | **Gene categories included** | **Observed** | **Null mean (10,000 permutations)** | **p** |
| --- | --- | --- | --- | --- |
| Strict | Canonical immune pathways | 1 | 1.08 | 0.61 |
| Broad | Strict set, plus proteostasis, autophagy, oxidative stress, and protease-related genes | 8 | 5.96 | 0.27 |

***Keyword sets (case-insensitive substring/regex matching against gene descriptions).***

| **Set** | **Category** | **Terms** |
| --- | --- | --- |
| Strict | Toll pathway | Toll, Spätzle, Cactus, Pelle, MyD88 |
| Strict | IMD pathway | IMD, Relish, Caspar, Dredd |
| Strict | JAK/STAT pathway | JAK, STAT, Hopscotch, Domeless, Unpaired |
| Strict | RNAi/antiviral | Argonaute, Dicer, Drosha, Loquacious, R2D2 |
| Strict | Melanization | Phenoloxidase, serpins, clip-domain serine proteases |
| Strict | Antimicrobial peptides | Lysozyme, defensin, cecropin, attacin, Gloverin, moricin, lebocin, diptericin, drosomycin, metchnikowin, gambicin |
| Strict | Pattern recognition | PGRPs, Gram-negative bacterial binding protein, β-1,3-glucan recognition protein, thioester-containing proteins, complement |
| Strict | Cellular immunity | Hemocyte, phagocytosis, encapsulation, nodulation, Nimrod, Eater, Draper, Croquemort, scavenger receptors |
| Strict | Other recognition/signaling | Dscam, cGAS-STING |
| Strict | General immune terms | Immune, immunity, antimicrobial, antiviral, antibacterial, defense response |
| Strict | Other immune-associated | Transferrin, ferritin, nitric oxide synthase, Hemolin, gamma-interferon-inducible thiol reductase |
| Broad (additional) | Proteostasis | Proteasome, ubiquitin, deubiquitinase |
| Broad (additional) | Autophagy | ATG genes, LC3, ULK, Beclin |
| Broad (additional) | Oxidative stress | Dual oxidase, NADPH oxidase, superoxide dismutase, catalase, peroxidase, peroxiredoxin, glutathione S-transferase, thioredoxin |
| Broad (additional) | Stress response | Heat shock proteins, molecular chaperones |
| Broad (additional) | Apoptosis | Caspases |
| Broad (additional) | Proteases/detoxification | Cytochrome P450s, carboxylesterases, serine proteases, trypsin, chymotrypsin |

**Figure S8. LD50 is not significantly correlated with individual life-history traits across inbred lines of Plodia interpunctella.** Each point represents one of the 12 inbred lines. Population-mean LD50 is plotted against **(A)** mean growth rate (mg day⁻¹), **(B)** mean pupal weight (mg), and **(C)** mean development time (days to pupation). Dashed lines show linear regression fits. None of the correlations are statistically significant: growth rate (r = -0.11, p = 0.73), pupal weight (r = -0.28, p = 0.35), and development time (r = -0.32, p = 0.29). The stock population is excluded from all panels. These results are consistent with the non-significant life-history PC1-LD50 correlation (Figure 5) and indicate that resistance was not detectably associated with the measured life-history traits across these inbred lines.

**Figure S9. K-means model selection for clustering of granulovirus resistance phenotypes across inbred lines of Plodia interpunctella.** K-means clustering was applied to population-mean LD50 values for k = 1 to 5, with 25 random starts per k (seed = 42). **(A)** Within-cluster sum of squares (WSS) as a function of k, showing a clear elbow at k = 3 beyond which additional clusters yield diminishing returns. **(B)** Percentage reduction in WSS relative to the previous k, showing high and stable improvement from k = 1 to k = 3 followed by a marked drop at k = 4. Red dashed lines indicate the selected value of k = 3, which is supported by both criteria. Clustering results are presented in Supplementary Figures S10 and S11.

**Table S7. K-means model-selection metrics for resistance clustering.**

K = 3 was selected based on the elbow in within-cluster sum of squares (WSS) and the marked reduction in incremental WSS improvement beyond k = 3. AIC and BIC are shown for completeness but continue to decrease through k = 5 and therefore do not independently identify k = 3 as an optimum.

| **k** | **WSS** | **% Improvement** | **AIC** | **BIC** |
| --- | --- | --- | --- | --- |
| 1 | 0.1732 | — | -48.86 | -48.38 |
| 2 | 0.0435 | 74.9% | -63.43 | -62.47 |
| 3 | 0.0105 | 75.9% | -78.53 | -77.08 |
| 4 | 0.0066 | 37.1% | -82.01 | -80.07 |
| 5 | 0.0040 | 39.4% | -86.05 | -83.62 |

**Figure S10. Distribution of LD50 values within resistance clusters identified by k-means clustering of Plodia interpunctella inbred lines.** Boxplots show the distribution of population-mean LD50 values for each of the three clusters identified by k-means clustering (k = 3) applied to LD50 values across the 12 inbred lines. Points represent individual inbred lines. Boxes show the interquartile range with the median as the central line. Clusters are color-coded by resistance level: blue = low resistance (n = 5 lines), gold = moderate resistance (n = 4 lines), red = high resistance (n = 3 lines). Cluster assignments were used to examine within-cluster relationships between resistance and life-history traits (Figure S11).

## **Table S8. Cluster assignments (k = 3)**

| **Population** | **LD50** | **Cluster** |
| --- | --- | --- |
| IL-7 | 0.811 | High resistance |
| IL-24 | 0.826 | High resistance |
| IL-2 | 0.754 | High resistance |
| IL-10 | 0.625 | Moderate resistance |
| IL-17 | 0.663 | Moderate resistance |
| IL-36 | 0.618 | Moderate resistance |
| IL-14 | 0.573 | Moderate resistance |
| IL-1 | 0.484 | Low resistance |
| IL-4 | 0.439 | Low resistance |
| IL-9 | 0.514 | Low resistance |
| IL-19 | 0.491 | Low resistance |
| IL-29 | 0.524 | Low resistance |

**Figure S11. Relationship between growth rate and granulovirus resistance within resistance clusters of Plodia interpunctella inbred lines.** Each point represents one inbred line, plotted by mean growth rate (mg day⁻¹) against population-mean LD50, with panels separated by resistance cluster assignment (see Figure S10). Lines show within-cluster linear regression fits. Pearson correlation coefficients and p-values are shown for each cluster. No within-cluster correlation is statistically significant at p < 0.05, though the low-resistance cluster approaches significance (r = -0.88, p = 0.051, n = 5), with higher growth rate associated with lower resistance. All within-cluster sample sizes are small (n = 3–5) and these analyses should be considered hypothesis-generating rather than confirmatory. Axes are standardized across panels to facilitate visual comparison among clusters.

**Figure S12**. **Window-level resistance-associated signal across genomic windows in *Plodia interpunctella* is more diffuse than expected under LD50 permutation.** Windows were ranked by PVE (r2) and cumulative share of summed window-level PVE was plotted against the fraction of windows included. These percentages summarize the distribution of window-level r^2^ values and are not an additive decomposition of phenotypic variance. The blue line shows the observed distribution across the 2,164 retained windows; the grey band shows the 95% null envelope generated by permuting LD50 across 12 lines 1,000 times and recomputing the PVE distribution under each permutation (dashed line: null mean). The observed cumulative PVE curve never exceeds the 95% null envelope at any rank and falls below its lower bound beyond approximately 24% of windows, indicating that window-level association strength is less concentrated among the highest-ranking windows and more broadly distributed across the genome than in the permuted datasets.

**Figure S13. Genomic PC1-LD50 association is similar under CDS-restricted and genome-wide variant sets.** Each point represents one of the 12 inbred lines, plotted by its score along the first principal component of genomic PCA against population-mean LD50, colored by LD50 (blue = susceptible, gold = intermediate, red = resistant). **(A)** CDS-restricted PCA (270,375 variants; r = -0.65, p = 0.022). **(B)** Genome-wide, complete-call-filtered PCA (1,717,334 variants; r = -0.57, p = 0.052). The solid line shows the linear regression fit with 95% confidence interval. The association is similar in direction and magnitude between the two variant sets, though the genome-wide analysis narrowly misses conventional significance, which may reflect dilution of the resistance-associated signal by a larger proportion of neutral genome-wide variation.
